# Neonatal near misses and associated factors among mother’s who give a live neonate at Hawassa City governmental hospitals, 2019: a facility based cross-sectional study design

**DOI:** 10.1186/s12884-021-03601-2

**Published:** 2021-02-12

**Authors:** Anteneh Fikrie Tekola, Genet Baye, Elias Amaje, Kebede Tefera

**Affiliations:** 1 Public Health Departement, Pharma College Hawassa Campus, Hawassa, Ethiopia; 2 College of Medical and Health Sciences, School of Public Health, Bule Hora University, Bule Hora, Ethiopia; 3 Project Officer, Bushulo Child Development and Family Strengthening Project, SOS Child Village Hawassa Program, Hawassa, Ethiopia; 4 College of Medicine and Health Sciences, School of Public Health, Hawassa University, Hawassa, Ethiopia

**Keywords:** Live neonates, Neonatal near miss, Governmental hospital, Hawassa City

## Abstract

**Background:**

Neonatal near miss is a neonate who nearly died but survived from a severe complication occurred during pregnancy, birth or within 0–28 days of extra-uterine life. However, there is no available data that quantifies the magnitude of neonatal near miss (NNM) in Ethiopia where there is high prevalence of neonatal mortality. Therefore, this study is designed to provide information about the magnitude and associated factors of neonatal near miss among women who give a live birth at Hawassa City Governmental hospitals, 2019.

**Methods:**

A facility based cross-sectional study design was conducted on 604 mothers who gave live neonates at Adare General Hospital and Hawassa University Comprehensive and Specialized Hospital from May 9, 2019 to June 7, 2019. Face to face interviewer administered structured questionnaire with a supplementation of maternal and neonatal medical records with checklists were used to collect the data. Data were coded and entered in to Epi data version 3.1 and then exported to the Statistical Package for Social Science IBM version 25 for analysis. Descriptive statistics was run and the data were presented using frequency tables and figure. The bi-variable and multivariable logistic regression was used to identify the possible factors of neonatal near miss. Finally, Adjusted Odds Ratio and 95% Confidence Intervals were used to declare statsticall significance.

**Result:**

Among all 604 selected live births an overall proportion of NNM cases, 202 (33.4%) (95% CI: 29.7–37.1%) was obtained at Hawassa City Government Hospitals. Respiratory distress 158 (94%) and infection or sepsis 138 (84%) were found to be the leading causes of NNM cases in our study. Governmental and non-governmental employed mother (AOR = 3.05, 95% CI: 1.46–6.44) and Cesarean Section delivery (AOR = 1.89, (95% CI: 1.25–2.83)) were positively significantly associated with neonatal near miss. Whereas, pregnancy induced Hypertension (AOR = 0.43, 95%CI: 0.27–0.69) was negatively associated with neonatal near miss.

**Conclusion:**

This study revealed relatively high prevalence of NNM in the study areas. Employed women, pregnancy induced hypertension and cesarean section mode of delivery were found to be independent factors affecting the prevalence of NNM cases. Therefore, HUCSH and Adare general Hospitals should focus on proving quality antenatal care and prevention of occupational related problems among pregnant women.

**Supplementary Information:**

The online version contains supplementary material available at 10.1186/s12884-021-03601-2.

## Background

Globally in 2017, 2.5 million babies died from preventable causes like prematurity, complication during the time of birth, bacterial infections, congenital malformations, and poor quality or no health care given at all. Almost all neonatal deaths (98%) occur in low- and middle-income countries, with a highest proportion of 78% in Southern Asia and sub-Saharan Africa. Eight of the 10 countries with the highest NMRs are in Africa [1, 2]. Neonatal mortality is still high in developing countries even if it is possible to minimize worldwide. It is estimated that the number of NNM is two to eight times higher than the number of neonatal deaths [3–5].

Ethiopia has achieved under-five mortality reduction Millennium Development Goal (MDG) targets [6]. However, both infant and neonatal mortality still remains a top priority plan of the national government because neonatal mortality rate shares the highest proportion 44% of under five deaths [7]. Neonatal mortality becomes continuous challenge for Ethiopia rolling from MDG to Sustainable Development Goal (SDG). The SDG target to end preventable neonatal deaths obliges all countries to reduce the neonatal mortality rate to 12/1000 live births by 2030 [1, 8].

According to Ethiopian Demographic and Health Survey (EDHS) 16 years report from 2000 to 2016, a steady declined infant, child and under-five mortalities were observed. Similarly, a 2016 EDHS report revealed a neonatal mortality rate of 29/1000 Live Birth (LB), meaning that 1 in every 35 neonates dies within the first month of their life [7]. Surprisingly, the 2019 mini EDHS report revealed an increased neonatal mortality, 30/1000LB, which is higher than prior report [9]. Different literatures show that NNM is a condition where newborns develop complication leading to nearly death condition but then survived in the first 28 days of life even though there is still no standard definition to classify NNM. Regardless of any criteria’s, neonatal deaths use as the gold standard [10, 11]. Thus, the best option to detect NNM cases in hospitals are intensive care units (ICUs) and neonatal ICUs combined with the pragmatic and management criteria [12]. Therefore, for this study, NNM criterions were adapted from World Health Organization (WHO) pragmatic and management criteria [11], Newborn Services Clinical Guideline, 2019 [2] and Ethiopian NICU guideline [13].

The main causes of neonatal death worldwide are complications arising from preterm birth, asphyxia during labor and sepsis, corresponding to 75% of these deaths. Majority of neonatal deaths are preventable and to reduce these deaths; invest in maternal and neonatal care during childbirth and in the first 24 h after birth [1, 2]. Severe infection, LBW and birth asphyxia were the most common causes of NNM in the health facilities [2, 14–16]. Similarly, in Ethiopia, 2015 government report revealed that Preterm birth complications 22%, intra-partum related events 32%, infection 20% and congenital abnormalities 12% were among the leading causes of neonatal death [2, 17, 18].

Adherence to essential newborn care would benefit newborns, adding special and intensive care services would reduce neonatal mortality by 50% [13]. Research conducted in Benchi Maji Zone revealed 22.8% of death among neonates who were admitted in NICU [19]. Identification and correction of factors that may improve maternal and neonatal care are more likely to contribute to the reduction in neonatal mortality rate [4]. Only few researches were conducted on neonatal near miss, even those studies that were conducted so far were unable to quantify the NNM cases in terms of its magnitude. Moreover, in the study setting there is no available data that quantifies the magnitude and factors associated with neonatal near miss.

Therefore, this study is intended to fill the gabs of those studies and to provide concrete information about the magnitude and associated factors of NNM in Hawassa City Governmental Hospitals.

## Methods

### Study setting, design and period

This hospital based cross-sectional study was conducted at Hawassa University Comprehensive Specialized Hospital (HU-CSH) and Adare General Hospitals which are both found at Hawassa City, Southern Ethiopia. Hawassa City is the capital city of southern nation’s nationalities and people’s region (SNNPR) from May 9, 2019 to June 7, 2019. HU-CSH is a teaching hospital for medicine and health sciences students. It has more than 350 beds, of which 84 are reserved for maternity beds, and perform more than 4378 deliveries per year. Likewise, Adare General Hospital has more than 126 beds, of which around 18 are used for maternity beds, and perform over 4238 deliveries per year. Both Hospitals are used as a referral where most complicated cases from the southern region and neighbor region zones served. Additionally, the city health centers are also referred the complicated cases to Adare General Hospital. These two hospitals are the only one giving ICU service for neonates at Zonal level for the catchment area.

### Study participants, inclusion and exclusion criteria

All live births during the data collection period at the selected Hospitals were included in the study, whereas multiple pregnancies, neonatal deaths, and neonates who were referred from other health care institutions that were out of the study hospitals were excluded.

### Sample size determination and sampling procedure

Sample size for first objective, assessing Neonatal Near Misses (NNM) was calculated using single population proportion formula. The specifications made during the computation were: Prevalence of NNM 36.7% [17], 95% confidence level, 4% margin of error and 10% compensation for possible missing values. The ultimate sample size was calculated as 614. Sample size for second objective was computed by Epi info7 Statcalc version 7.1.4.0 software by the assumptions of, 95% level of confidence, power of 80%, the ratio of exposed to unexposed 1:1 and percent of outcome in unexposed group 15.9 and AOR of 2. The percent of outcome in unexposed group and AOR were taken from the study conducted in Southern Ethiopia; the determinate variable was premature rupture of membrane [17]. By substituting the above values in to software the estimated sample size was 432. By comparing the two sample size calculated, the first sample size was larger than the second as a result we took 614 as the final calculated sample size for the study. Subsequently, the calculated sample size was allocated for both hospitals proportionally based on their prior annual delivery report. Subsequently, due to the rare cases of NNM, all the consecutive live births were included in the study during the study period.

#### Data collection and quality assurance

Data were collected by a face-to-face-interviewer administered structured questionnaire and standard data extractions checklist from medical record were used to collect the data. The standard data extraction was prepared by reviewing prior literatures [12, 16, 20] and WHO recommended information [11]. As there are different languages in the study area, Hawassa City, the questionnaire was primarily prepared in English and translated to the regional working language, Amharic, during the interview (additional file 1). Four diploma nurses and two Degree holder Nurses were recruited as the data collectors and supervisors respectively. All questions in the questionnaire were clarified to each data collectors before the data collection period. Likewise, the data collectors were trained on how to ask questions exactly as stated in the questionnaire and provide only non-directive guidance. Following to three days training, data collectors started the data collection. To reduce information bias the questionnaire was pretested on Bushulo Maternity Health Center prior to the actual data collection period. Primary data, socio-demographic and economic characteristics of mothers, were collected through face-to-face-interview and secondary data, obstetrics and medical history of mothers and neonatal characteristics, were extracted from maternal and neonate medical records by standard checklist.

Neonates who sustained NNM cases were identified by well trained and experienced data collectors using the standard WHO recommended pragmatic or management severity criteria’s (Table 1). Independent variables were: Socio-economic and demographic characteristics (Age, income, Household size, maternal and paternal educational status, place of residence, maternal occupational status, marital status), maternal obstetric history (ANC, frequency of ANC, parity, gravidity, gestational age at first ANC visit, abortion history, Premature Rupture of Membrane, Mode of delivery) and maternal medical history (Diabetic mellitus, Pregnancy induced diabetic mellitus, Anemia, Hypertension, Pregnancy induced hypertension, syphilis).

**Table 1:** Criteria to identify neonatal near-miss cases. This Table shows the criteria to identify neonatal near-miss cases in HU-CSH and Adare General Hospitals, Hawassa City, Southern Ethiopia, 2019

Criteria	Descriptions
Pragmatic criteria	Birth weight less than 1750 g, Gestational Age less than 33 weeks and Apgar score less than 7 at 5 min [11, 21]
Management severity criteria	Respiratory distress, blood transfusion, presence of infection with clinical concern, Bile stained vomiting, feeding problems severe enough to cause clinical concern, Cardiopulmonary resuscitation, Congenital Malformations, Convulsion, Surgery, Phototherapy within 24 h of life, Parenteral intravenous drugs or nutrition, Any intubation [11, 21].

#### Data processing and analysis

Data were checked for completeness and consistencies, coded and entered into Epi data version 3.1 then exported to Statistical Package for Social Science (SPSS) version 25 for analysis. Continuous variable, maternal age was summarized by median with IQR because the data were not normally distributed and presented using frequency tables, figures and charts. The bi-variable and multivariable logistic regression was used to identify the possible factors of neonatal near miss at Hawassa city governmental hospitals. A variable with *p* value ≤0.2 during bivariate analysis was entered in to multivariable logistic regression for further analysis so as to control the confounding variables. Multi co-linearity was checked by collinearity statistics (Variance inflation factor). Finally, Adjusted Odds Ratio (AOR) and 95% Confidence Intervals (CIs) were used to declare statstical significance.

## Results

### Socio-demographic characteristics of respondents

During the study period, a total of 724 live births attended in both study hospitals. Of these, 604 records were met the eligibility criteria and included in this study and made the response rate of 98.3%. Whereas, of which the eligibility of 10 (1.7%) could not be included because of incomplete maternal data records at least with one variable. The median (±IQR) age of the respondents was 26 (±7) years with a minimum and maximum age of 15 and 49 years respectively. Majority of the respondents 364 (60.3%) fall in the 25–34 years’ age group. The vast majority of neonate’s mothers 568 (94%) were married. Regarding the occupational status of respondents more than half of respondents, 317 (52.5%) were housewives. Out of the total respondents, 88 (14.6%) of the study participants had monthly income of less than 43 USD. Pertaining to educational status of the respondents, 86 (14.2%) never attended any type of formal education (Table 2).

**Table 2:** Socio-demographic characteristics of mothers. This table indicates the socio-demographic characteristics of mothers who gave live birth in Hawassa City Governmental Hospitals, Southern Ethiopia, 2019(*n* = 604)

Characteristics	Frequency	Percent (%)
Age in years		
15–24	191	31.6
25–34	364	60.3
≥ 35	49	8.1
Marital status		
Married	568	94
Others*	36	4
Place of residence		
Urban	437	72.4
Rural	167	27.6
Occupation		
House wife	317	52.5
Employed	144	23.8
Merchant	42	7.0
Others^#^	101	16.7
Maternal Education		
Illiterate	86	14.2
Primary education	195	32.3
Secondary education	161	26.7
College and above	162	26.8
Father’s Education		
Illiterate	47	7.8
Primary education	127	21.0
Secondary education	171	28.3
College and above	259	42.9
House hold family size		
< 4	331	54.8
4–7	213	35.3
> 7	60	9.9

*single, divorced ^#^farmers, students, daily labors

#### Maternal obstetrics and chronic disease history characteristics

Of all the neonates’ mother, 548 (90.7%) had at least one ANC follow up during their current pregnancy. On the other hand, almost half, 291 (48.2%) of the respondents had less than four ANC visits in their current pregnancy. More than two third, 420 (69.5%) of respondents were multigravida. Regarding the parity, 250 (41.4%) of the respondents were multiparous. One in seven, 89 (14.7%) and one in ten, 59 (9.8%) respondents had history of abortion and neonatal deaths respectively. Regarding the mode of delivery, the majority 397 (65.7%) gave birth by vaginal deliveries. More than half of respondents, 340 (56.3%) had premature rupture of membrane.

Regarding chronic diseases history of respondents, 99 (16.4%), 11 (1.8%) and 7 (1.2%) had pregnancy induced hypertension, Chronic Diabetes mellitus and pregnancy induced diabetic mellitus during the current pregnancy respectively. Similarly, among all neonates’ mothers, 13 (2.2%) had diagnosed with syphilis during the current pregnancy. The vast majority, 553 (91.6%) of the respondents had vaginal bleeding (Table 3).

**Table 3:** Obstetric and chronic disease characteristics of mothers This table shows the obstetric and chronic disease characteristics of mothers at Hawassa City Government Hospitals, Southern, Ethiopia, 2019 (*n* = 604)

Variable (*n* = 604)	Frequency	Percent (%)
ANC		
Yes	548	90.7
No	56	9.3
Parity		
Nulliparous	182	30.1
Primiparous	172	28.5
Multiparous	250	41.4.
History of abortion		
Yes	89	14.7
No	515	85.3
Number ANC (*n* = 548)		
< 4	291	48.2
> =4	257	42.5
Gestation at first visit (n = 548)		
First trimester	173	28.6
Second trimester	338	56
Third trimester	37	6.1
Premature rupture of membrane		
Yes	340	56.3
No	264	43.7
Mode of delivery		
Vaginal deliveries*	397	65.7
Cesarean section	207	34.3
Pregnancy induced hypertension		
Yes	99	16.4
No	505	83.6
Diabetic mellitus		
Yes	11	1.8
No	593	98.2
Hypertension		
Yes	46	7.6
No	558	92.4
Pregnancy induced diabetic mellitus		
Yes	7	1.2
No	597	98.2
Syphilis		
Yes	13	2.2
No	591	97.8
Vaginal bleeding		
Yes	553	91.6
No	51	8.4

#### Neonatal near miss

Among all 604 selected live births an overall proportion of NNM cases, 202 (33.4%; 95% CI: 29.7–37.1%) was observed. In this study the majority of neonates were tackled by respiratory distress, 158 (78.2%) followed by sepsis 138 (68.3%). Seven (3.46%) of NNM cases were admitted due to the need of blood transfusion (Table 4).

**Table 4:** Prevalence of NNM cases based on pragmatic and management criteria’s. This table indicates the prevalence of NNM cases based on pragmatic and management criteria’s at Hawassa City Government Hospitals, Southern Ethiopia, 2019 (*n* = 202)

Criteria’s		Neonatal Near Miss cases	
		Number	Percent (%)
Pragmatic criteria’s	Birth weight < 1750	11	5.44
	Gestational age < 33 weeks	24	11.88
	APGAR^*^ score < 7 at 5th minute	56	27.72
Management criteria’s	Respiratory distress	158	78.21
	Infection	138	68.31
	Parenteral nutrition	170	84.15
	Blood transfusion	7	3.46
	Bile stained vomiting	14	6.93
	Feeding problems	82	40.59
	Cardiovascular Problems	14	6.93
	Congenital Malformations	17	8.41
	Convulsion	9	4.45
	Surgery	11	5.44
	Phototherapy within 24 h of life	53	26.23
	Any intubation	83	41.09

^*^APGAR: Appearance, Pulse, Grimace, Activity, and Respiration

#### Factors associated with NNM

After adjusting or controlling the potential confounding variables by the multivariate analysis: Employed mothers [AOR = 3.05(95% CI: 1.46–6.44)], pregnancy induced HTN [AOR = 0.42(95%CI: 0.26–0.66)] and cesarean mode of delivery [AOR = 1.89(95% CI:1.25–2.83)] were significantly associated with NNM cases. Accordingly, the odds of NNM were three fold among neonates whose mothers occupational status is employed as compared to their counterparts (AOR = 3.05, 95% CI: 1.46–6.44). A mother who had pregnancy induced HTN was 58% times less likely to have NNM neonate as compared to mothers hadn’t (AOR = 0.42, 95%CI: 0.26–0.66). A neonate’s mother who gave birth by cesarean mode of delivery were nearly 1.9 times more likely to have NNM cases compared to mothers who gave birth through vaginal deliveries (AOR = 1.89, 95% CI:1.25–2.83) (Table 5).

**Table 5:** Factors associated with neonatal near miss. This table shows factors associated with neonatal near miss among mothers giving live births at Hawassa City government Hospitals, Southern, Ethiopia, 2019 (*n* = 604)

Variable	Neonatal Near miss		COR (95% CI)	AOR (95% CI)
	Yes N (%)	No N (%)		
Age in years				
15–24	65 (32.2)	126 (31.3)	1	
25–34	120 (59.4)	244 (60.7)	1.04 (0.72–1.51)	
≥ 35	17 (8.4)	32 (8)	0.97 (0.50–1.87)	
Marital status				
Married	190 (94.1)	378 (94)	0.99 (0.48–2.03)	
Others^**&**^	12 (5.9)	24 (6)	1	
Maternal Education				
Illiterate	42 (20.8)	44 (10.9)	0.42 (0.25–0.73)	0.65 (0.30–1.41)
Primary education	68 (33.7)	127 (31.6)	0.76 (0.48–1.19)	1.39 (0.72–2.69)
Secondary education	45 (22.3)	116 (28.9)	1.05 (0.65–1.71)	1.68 (0.89–3.17)
College and above	47 (23.3)	115 (28.6)	1	1
Occupational status of mothers				
House wife	117 (57.9)	200 (49.8)	1.16 (0.73–1.84)	1.56 (0.92–2.63)
Employed (Gov’t, NGO & Private)	33 (16.3)	111 (27.6)	2.29 (1.31–4.00)	3.05 (1.46–6.34)*
Merchant	11 (5.4)	31 (7.7)	1.92 (0.87–4.26)	2.26 (0.95–5.35)
Others^#^	41 (20.4)	60 (14.9)	1	1
Father’s Education				
Illiterate	17 (8.4)	30 (7.5)	1	
Primary education	57 (28.2)	70 (17.4)	0.69 (0.43–1.38)	
Secondary education	51 (25.2)	120 (29.9)	1.33 (0.67–2.63)	
College and above	77 (38.1)	182 (45.3)	1.33 (0.69–2.57)	
House hold family size				
< 4	110 (54.5)	221 (55)	1	1
4–7	66 (32.7)	147 (36.6)	1.10 (0.76–1.60)	1.27 (0.85–1.90)
≥ 7	26 (12.9)	34 (8.9)	0.65 (0.37–1.13)	0.97 (0.51–1.85)
Residence				
Urban	147 (72.8)	290 (72.1)	1	
Rural	55 (27.2)	112 (27.9)	1.03 (0.70–1.50)	
ANC Visit				
Yes	182 (90.1)	366 (91)	1	
No	20 (35.7)	36 (64.3)	0.89 (0.50–1.59)	
Number of ANC				
No visit	20 (9.9)	36 (9)	1	1
1–3	111 (55)	180 (44.88)	0.90 (0.49–0.164)	0.63 (0.31–1.27)
≥ 4	71 (35.1)	186 (46.12)	1.44 (0.78–2.66)	1.02 (0.48–2.16)
Vaginal bleeding				
Yes	181 (89.6)	372 (92.5)	1.44 (0.80–2.58)	1.46 (0.77–2.76)
No	21 (10.4)	30 (7.5)	1	1
Mode of delivery				
Vaginal	113 (55.9)	284 (70.6)	1	**1**
Cesarean section	89 (44.1)	118 (29.4)	1.90 (1.33–2.69)	1.89 (1.25–2.83)**
Parity				
Nulliparous	65 (32.2)	117 (29.1)	0.97 (0.65–1.45)	
Primiparous	49 (24.3)	123 (30.6)	1.36 (0.89–2.00)	
Multiparous	88 (43.6)	162 (40.3)	1	
Rupture of membrane				
Yes	103 (51)	237 (59)	1.38 (0.98–1.94)	1.19 (0.78–1.81)
No	99 (49)	165 (41)	1	1
Hypertension				
Yes	17 (8.4)	29 (7.2)	0.84 (0.45–157)	
No	185 (91.6)	373 (92.8)	1	
Pregnancy induced HTN				
Yes	52 (25.7)	47 (11.7)	0.38 (0.24–0.59)	0.42 (0.26–0.66)***
No	150 (74.3)	355 (88.3)	1	**1**

COR:Crude Odds Ratio, AOR: Adjusted Odds Ratio, HTN:Hypertension, ANC: Antenatal Care, *** *P*-value < 0.0001, ** *p*-value < 0.001, * *p*-value < 0.05: ^**&**^Single, divorced, widowed: ^**#**^student, daily labors, and farmers

## Discussion

Among all 604 selected live births an overall proportion of NNM cases, 202 (33.4%) [95% CI: 29.7–37.1%] was obtained at Hawassa City Government Hospitals. These NNM cases were identified using pragmatic or management criteria’s alone or combined. Occupational status of mothers, Pregnancy induced Hypertension and mode of delivery were found to be independent predictors of factors affecting the prevalence of NNM cases among mothers who gave live neonates at Hawassa City Governmental Hospitals.

The finding of this study revealed that NNM cases of 33.4%, which is ten times higher than the national neonatal death rate [9]. The result of this study is in line with similar other study conducted in Northeastern Brazil [12] and Uganda [16]. However, the finding of this study was inconsistent with similar studies conducted worldwide: low and middle income countries in Africa [20] and three similar studies in Brazil [3, 5, 14]. The observed high prevalence in this study might be due to the referral nature of the health institutions where an increased probability of attending more complicated cases from the catchment area and neighboring region. Moreover, the hospital delivery rate, which is 50% [9], in Ethiopia, might be another contributing factor to the observed high rate in this study compared to other studies.

According to this study, cesarean section identified as a risk factor for NNM. Thus, the odds of NNM cases were 1.9 times higher among mothers who gave birth through cesarean section as compared to mothers who gave birth through vaginal deliveries (AOR = 1.89, (95% CI:1.25–2.83)). This is result is corroborated by different studies conducted at national and global level [3, 4, 10, 17]. The possible reason for this is that cesarean section delivery has increased Neonatal morbidity and mortality and also result in slow or no improvement of neonatal outcomes [22]. Also, cesarean section delivered newborns had less skin-to-skin contact with their mothers immediately after delivery and makes the neonates unable to breastfeeding within one hour of birth in turn put the neonate at greater risk of early complications [23]. Likewise cesarean section on demand sometimes could be a risk factor for prematurity, which is one of the components of pragramatic criteria. However, the cesarean section delivery by itself could not be responsible for adverse neonatal outcomes. Rather the occurrences of maternal and fetal complications which would lead to indication of cesarean section could actually be responsible for the incident of neonatal near miss. Therefore, cesarean section should be undertaken merely owing to medical indication.

The current study shows that neonate’s mothers who have pregnancy induced HTN had 58% times reduced odds of experiencing neonatal near miss as compared to mothers who had no pregnancy induced hypertension (AOR = 0.42, 95% CI: 0.26–0.66). This result is corroborated by a study conducted at Brazil [24]. This congruency might be due to the fact that women with gestational hypertension are more likely to have higher rates of CS delivery so as to prevent neonatal complications leading to NNM cases early. There is also scientific evidence about the importance of use of CS during delivery among women with pregnancy induced hypertension [25]. However, this study result is incongruent with a study conducted in Southern Ethiopia [17], the reason might be methodological (study area, sample size and study design) difference.

There is published evidence for the adverse effects of occupational stress on fetal growth and development [26]. This study also revealed that the odds of NNM cases were three times higher among employed mothers than unemployed mothers (AOR = 3.05, 95% CI: 1.46–6.44). This finding is consistent with other previous studies conducted in abroad [26–28]. This is due to the fact that women who are engaged in hard physical work are at most risk to have adverse pregnancy outcomes [29]. Furthermore, women who are working in unfavorable environments such as; stress, prolonged standing and sitting, and contact with different chemicals could expose her to have a preterm birth and neonatal abnormalities, which are the predominant causes of NNM [28].

This study has added weight to the existing literature by quantifying the NNM cases and might be used as an input for health policymakers and program developers particularly working on neonatal health in the health care delivery system. However, the findings from this study would be difficult to extrapolate to the wider population, because the study was hospital based, but not community based. Data were only collected at appoint in time from mothers who gave live newborns at the hospitals and, therefore, cases referring from the community may have been missed. The prevalence of NNM might be higher because HUCSH is tertiary type of hospital and Adare general hospital is also a referral option for all government and private health facilities for Hawassa city, Sidama and neighborhoods zones this might increase the possibility of attending more complicated cases resulting over estimation of the magnitude. Moreover, the data were collected with the supplementation of medical records, which were incomplete of some variables for NNM like neonatal weight and Apgar score, and as a result they were obliged to be excluded. The participants were recruited during only one month and unable to consider seasonality as a risk factor of neonatal mortality. A prospective longitudinal research should be conducted by incorporating the full 28-days of the postnatal period.

## Conclusions

In this study a higher prevalence of NNM cases was obtained at Hawassa City Government Hospitals. Cesarean Section delivery, employed mother and pregnancy induced hypertensions were significantly associated with NNM in this study. Respiratory distress and sepsis were also identified as the leading causes of NNM cases. Focused antenatal care should be strengthened so as to prevent the complications early. Likewise, an emphasis should also be given to pregnant women who are employed to government or private organizations to make sure that they are free from occupational related problems. The STROBE guideline was used to ensure the reporting of this cross-sectional study (Additional file 2).

## Supplementary Information

**Additional file 1.** English version questionnaire.

**Additional file 2.** STROBE Checklist for reporting cross-sectional studies.

## Data Availability

Data essential for the conclusion are included in this manuscript. Additional data can be obtained from the corresponding author on a reasonable request.

## Abbreviations

ANC: Ante Natal Care
AOR: Adjusted Odds Ratio
CI: Confidence Interval
COR: Crude Odds Ratio
CS: Cesarean Section
EDHS: Ethiopian Demographic and Health Survey
ICU: Intensive care unit
IQR: Inter Quartile Range
LB: Live Birth
LBW: Low Birth Weight
NICU: Neonatal Intensive Care Unit
NMR: Neonatal Mortality Rate
NNM: Neonatal Near Miss
SDGs: Sustainable Development Goals
WHO: World Health Organization
