# Supplementary material for: Neonatal near misses and associated factors among mother’s who give a live neonate at Hawassa City governmental hospitals, 2019: a facility based cross-sectional study design
Source: BMC Pregnancy Childbirth. 2021 Feb 12;21:125. doi: 10.1186/s12884-021-03601-2 (PMC7881473; doi:10.1186/s12884-021-03601-2)
Supplement: Supplementary file 1 — Additional file 1. English version questionnaire. [file 12884_2021_3601_MOESM1_ESM.pdf]

Neonatal near misses and associated factors among mother's who give a live neonate at Hawassa City Governmental Hospitals, 2019. A facility based Cross-sectional study design

Anteneh Fikrie Tekola[1, 3*], Genet Baye[2], Elias Amaje[3] and Kebede Tefera[4]

[1] Public Health Departement, Pharma College Hawassa Campus, Hawassa, South Ethiopia,

[2] Project Officer, Bushulo Child Development and Family Strengthening Project, SOS Child Village Hawassa Program, Hawassa, South Ethiopia,

[3] School of Public Health, College of Medical and Health Sciences, Bule Hora University, Bule Hora, South Ethiopia,

[4]School of Public Health, College of Medicine and Health Sciences, Hawassa University, Hawassa, South Ethiopia,

***** Corresponding authorEmail: antenehfikrie3@gmail.com

ENGLISH VERSION QUESTIONNAIRE

Questionnaire number ____________________ Date of interview________________________				
Name of interviewer_______________________ Name of Supervisor____________________				
**Section I: socio-economic and demographic data**				
s.no	Question	Option	Skip	Code
101	How old are you?	---------------- years		
102	Marital status	1. Single<br>2. Married<br>3. Divorced<br>4. Widowed<br>5. Separated		
103	Place of residence?	1. Urban<br>2. Rural		
104	Maternal Educational status	1. Illiterate<br>2. Primary school (grade 1-8)<br>3. Secondary (grade 9-12)<br>4. Twelve and above (12+)		
105	Paternal Educational status	1. Illiterate<br>2. Primary school (grade 1-8)<br>3. Secondary (grade 9-12)<br>4. Twelve and above (12+)		

106	Maternal occupational status	1. Farmer<br>2. house wife<br>3. student<br>4. Government employee<br>5. private organization employee<br>6. NGO employee<br>7. merchant<br>8. Daily laborer<br>9. others, specify____________		
107	Average monthly income	-------------- birr		
108	Family size	---------------in number		
**Section II: obstetrics history of the mother**				
201	How many times do you get pregnant including the current? (gravidity)	-------------times		
202	How many of your pregnancy were >28 weeks?(parity)	-------------times		
203	Have you ever had an abortion? (<28 weeks)if yes how many ?	1. Yes-----------<br>2. No		
204	How many of your children are alive currently?	-------------		
205	Did you lose a baby within 28 days afterbirth?	1. Yes<br>2. No		
206	Do you have ANC visit ever for the current pregnancy?	1. Yes<br>2. No		
207	How many months pregnant were you at first visit of ANC for the current pregnancy?	------------months		
208	Was your weight measured during ANC visit?	1. Yes------------Kg<br>2. No		
209	Was your blood pressure measured during ANC visits	1. Yes<br>2. No		
210	Have you done all the lab examinations during ANC of the current pregnancy? (observation of medical record)	1. Blood<br>2. Urine<br>3. Stool<br>4. Others		
211	How many times did you have ANC visits throughout your current pregnancy?	-----------times		
212	How many months pregnant were you when you gave birth of the current pregnancy?	------------------months		

213	Have you ever told about any danger sign of complication?	1. Yes<br>2. No	If no - 215	
214	Which danger sign of pregnancy were you told?	(yes / N0)<br>Vaginal bleeding 1 2<br>Severe headache 1 2<br>Blurred vision 1 2<br>Vaginal gush of Fluid 1 2<br>Fever 1 2<br>Abdominal pain 1 2<br>Other (specify):		
215	Did you take iron and folate during this pregnancy?	1. Yes<br>2. No	If no-217	
216	If yes, how long?	------------------		
217	Have you been told that you have anemia for the current pregnancy?	1. Yes<br>2. No		
218	If yes have you been treated?	1. Yes<br>2. No		
219	How long did you stay home after your water broke for the current pregnancy? (rupture of membrane)	1. 2 days<br>2. 1.5 day<br>3. 1day<br>4. Immediately<br>5. Specify -----------		
220	When did you decide to come to hospital after as current labour starts? (decision time)	1. Immediately<br>2. Half day<br>3. One day<br>4. Specify--------		
221	Did you get transport access to reach to hospital as you decided to get health care service? (how long did it take to reach hospital)	1. Immediately<br>2. One-hour after<br>3. Half day<br>4. Other specify -----------		
222	Have you got health care service immediately as you reach the hospital?	1. Yes<br>2. No	If yes skip 223	
223	If no how long did you stay before getting service? Specify	------------------		
**Section III: Maternal medical history**				
301	Did you take any drug for intestinal parasites during the	1. Yes<br>2. No		

	current pregnancy?	3. Do not remember		
302	Have you ever diagnosed severe infection leading to admission or Iv drugs during this pregnancy?	1. Yes<br>2. No<br>3. Don't remember		
303	Did you have Hypertension diagnosed by health professionals before this pregnancy?	1. Yes<br>2. No		
304	Did you have Hypertension developed after getting this pregnancy?	1. Yes<br>2. No		
305	Did you have Diabetic Mellitus diagnosed before this pregnancy?	1. Yes<br>2. No		
306	Did you have Diabetic Mellitus developed after getting this pregnancy?	1. Yes<br>2. No		
307	Have you been diagnosed for syphillis during the current pregnancy?	1. Yes<br>2. No		
308	Is there any other chronic disease diagnosed before? If there is, specify.	__________________________		
**Section IV: Maternal behavioral or lifestyle history**				
401	Have you been cooking with smoke during this pregnancy?	1. Yes 2. No	If no- 404	
402	If yes, how frequent?	1. Always 2. Sometimes		
404	Did you consume alcohol drinks during this pregnancy?	1. Yes 2. No		
405	If yes, how frequent did you take alcohol drinks?	1. Daily<br>2. 3 times per week<br>3. 1 times per week<br>4. Once a month		
406	Did you smoke cigarrate during this pregnancy?	1. Yes<br>2. No		
407	If yes, how frequent did you smoke?	1. Daily<br>2. 3 times per week<br>3. 1 times per week<br>4. Once a month		
408	Did you chew chat during this pregnancy?	1. Yes<br>2. No		
409	If yes, how frequent did you chew chat?	1. Daily 2). 3 times per week<br>3). 1 times per week 4). 1 per month		

**Section V:Condition of the Neonate**				
501	Mode of delivery of the current pregnancy	1.Vaginal delivery<br>2.Assisted delivery<br>3.Cesarean section		obser vatio n
502	Gestational age of neonate at birth	----------weeks		
503	Neonates gender	1. Male 2. Female		
504	Neonates weight(in grams)	-------------grams		
505	Identified problem either pragmatic or management criteria (circle it)<br><br>These criteria's will be selected by the interviewer using health records and observation of the neonate More than one choice is possible.	**Pragmatic criteria(checklist)**	Yes	No
		1. Birth weight less than 1750 g		
		2. Apgar score less than 7 at 5th minute		
		3. Gestational age less than 33weeks		
		Management criteria's	Yes	No
		4. Respiratory distress/apnea		
		5. Blood transfusion		
		6. Presence of infection		
		7. Persisting signs of respiratory distress for more than one hour.		
		8. Requirement for intermittent positive pressure ventilation.		
		9. Bile stained vomiting (signs suggesting bowel obstruction)		
		10. Feeding problems severe enough to cause clinical concern		
		11. Cardiovascular Problems requiring monitoring or intervention		
		12. Congenital Malformations that may require intervention		
		13. Convulsion		
		14. Surgery		
		15. Phototherapy in 24 hours of life.		
		16. parenteral intravenous drugs or nutrition		
		17. Any intubation		
506	One of the above criteria's present, classify as neonatal near miss case			
507	If there is no problem identified among list of question number 505,then			

	classify as normal neonate			
