# Supplementary material for: Neonatal near misses and associated factors among mother’s who give a live neonate at Hawassa City governmental hospitals, 2019: a facility based cross-sectional study design
Source: BMC Pregnancy Childbirth. 2021 Feb 12;21:125. doi: 10.1186/s12884-021-03601-2 (PMC7881473; doi:10.1186/s12884-021-03601-2)
Supplement: Supplementary file 2 — Additional file 2. STROBE Checklist for reporting cross-sectional studies. [file 12884_2021_3601_MOESM2_ESM.pdf]

STROBE Statement—Checklist of items that should be included in reports of ***cross-sectional studies***			
	**Item No**	**Recommendation**	**Remarks**
**Title and abstract**	1	(*a*) Indicate the study's design with a commonly used term in the title or the abstract	"Title page, line 3"
		(*b*) Provide in the abstract an informative and balanced summary of what was done and what was found	"Abstract, page number 2"
**Introduction**			
Background/rationale	2	Explain the scientific background and rationale for the investigation being reported	"Background, paragraph 5"
Objectives	3	State specific objectives, including any prespecified hypotheses	"Background, paragraph 5 line 8-10"
**Methods**			
Study design	4	Present key elements of study design early in the paper	"Methods, paragraph 1"
Setting	5	Describe the setting, locations, and relevant dates, including periods of recruitment, exposure, follow-up, and data collection	"Methods, paragraph 1"
Participants	6	(*a*) Give the eligibility criteria, and the sources and methods of selection of participants	"Methods, Study participants, inclusion and exclusion criteria section"
Variables	7	Clearly define all outcomes, exposures, predictors, potential confounders, and effect modifiers. Give diagnostic criteria, if applicable	"Methods, data collection and quality assurance section paragraph 2"
Data sources/ measurement	8*	For each variable of interest, give sources of data and details of methods of assessment (measurement). Describe	"Methods, data collection and quality assurance section paragraph 1"

		comparability of assessment methods if there is more than one group	
Bias	9	Describe any efforts to address potential sources of bias	"Methods, data collection and quality assurance section paragraph 1"
Study size	10	Explain how the study size was arrived at	"Methods, Sample size determination and sampling procedure section"
Quantitative variables	11	Explain how quantitative variables were handled in the analyses. If applicable, describe which groupings were chosen and why	"Methods,Data processing and analysis section line 3"
Statistical methods	12	(*a*) Describe all statistical methods, including those used to control for confounding	"Methods,Data processing and analysis section line 7"
		(*b*) Describe any methods used to examine subgroups and interactions	"Methods,Data processing and analysis section line 9"
		(*c*) Explain how missing data were addressed	"N/A".
		(*d*) If applicable, describe analytical methods taking account of sampling strategy	"Methods,Data processing and analysis section line 4"
		(*e*) Describe any sensitivity analyses	"N/A".
**Results**			
Participants	13*	(a) Report numbers of individuals at each stage of study—eg numbers potentially eligible, examined for eligibility, confirmed eligible, included in the study, completing follow-up, and analysed	"Results, paragraph 1 line 1"
		(b) Give reasons for non-	"Results, paragraph 1 line 4"

		participation at each stage	
		(c) Consider use of a flow diagram	"N/A".
Descriptive data	14*	(a) Give characteristics of study participants (eg demographic, clinical, social) and information on exposures and potential confounders	"Results, table 2 and 3"
		(b) Indicate number of participants with missing data for each variable of interest	"N/A".
Outcome data	15*	Report numbers of outcome events or summary measures	"Neonatal Near miss, page 8"
Main results	16	(*a*) Give unadjusted estimates and, if applicable, confounder-adjusted estimates and their precision (eg, 95% confidence interval). Make clear which confounders were adjusted for and why they were included	"Factors associated with NNM, page 8 and Table 5 page 21"
		(*b*) Report category boundaries when continuous variables were categorized	"Factors associated with NNM, page 8 and Table 5 page 21"
		(*c*) If relevant, consider translating estimates of relative risk into absolute risk for a meaningful time period	"N/A".
Other analyses	17	Report other analyses done—eg analyses of subgroups and interactions, and sensitivity analyses	"N/A".
**Discussion**			
Key results	18	Summarise key results with reference to study objectives	"Discussion, paragraph 1"
Limitations	19	Discuss limitations of the study, taking into account sources of potential bias or imprecision.	"Discussion, paragraph 6"

		Discuss both direction and magnitude of any potential bias	
Interpretation	20	Give a cautious overall interpretation of results considering objectives, limitations, multiplicity of analyses, results from similar studies, and other relevant evidence	"Discussion, paragraph 2-5"
Generalizability	21	Discuss the generalizability (external validity) of the study results	"Discussion, paragraph 6"
**Other information**			
Funding	22	Give the source of funding and the role of the funders for the present study and, if applicable, for the original study on which the present article is based	"Declarations, funding section"
